# Granulosis Rubra Nasi: A Case Report and Brief Review of the Literature

**DOI:** 10.1155/2023/3927244

**Published:** 2023-01-27

**Authors:** Girum T. Assefa

**Affiliations:** Department of Dermatovenereology, College of Medicine and Health Science, Hawassa University, Hawassa, Ethiopia

## Abstract

Granulosis rubra nasi is a rare autosomal dominant disease of the eccrine glands. It is clinically characterized by mid-face hyperhidrosis, most prominent at the tip of the nose, and dark erythematous papules on the nose, cheeks, chin, and upper lip. Although it commonly occurs in childhood, it can also occur in adults. This is a case report of two female granulosis rubra nasi patients. This report, to the best of my knowledge, has not before been described in Ethiopian individuals and is hence being reported due to its rarity.

## 1. Introduction

Granulosis rubra nasi (GRN), also known as “Acne papulo-rosacea of the nose,” is a childhood inflammatory disorder of the eccrine glands first described by German dermatologist Jadassohn in 1901 [1].

It is an autosomal dominant familial disease of children involving the eccrine glands of the nose, cheeks, and chin. This condition appears to be extremely rare [2] and generally appears during childhood, but adolescent or adult onset is possible [3]. The etiology and pathogenesis of this disorder have not been elucidated. The term is sometimes used as a diagnostic title for hyperhidrosis of the nose [2].

It is a chronic condition with a benign outcome. The cause is unknown and usually goes away on its own during adolescence. The treatment is both symptomatic and cosmetic in nature.

I am reporting 2 cases of GRN here.

## 2. Case Report

**Figure 1:**
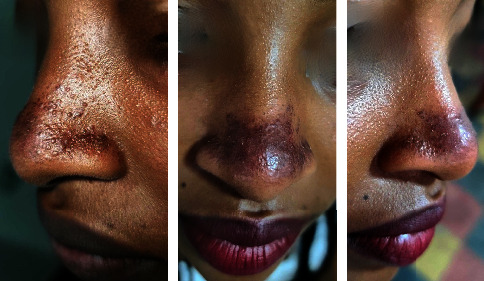
GRN: Discrete dark erythematous papule over the alae and violaceous erythema over the tip of the nose.

The first case is a 19-year-old female who presented with a raised red lesion over the nose since childhood without sensitivity to sunlight. She was otherwise well, and there was no relevant family history. Physical examination revealed multiple dark erythematous papules in the nasal dorsum and alae with violaceous patches over the apex (Figure 1). The rest of the physical examination was normal. The patient was treated with topical metronidazole and sunscreen at another medical centre and had no improvement.

**Figure 2:**
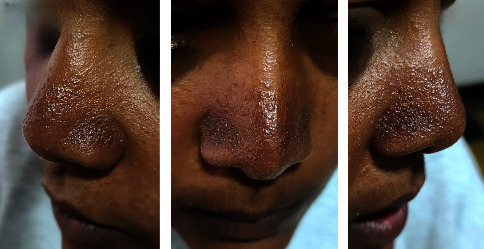
GRN: Discrete multiple erythematous papule over the tip and alae of the nose.

The second patient is a 25-year-old female who presented with redness and excessive sweating over the nose for more than 5 years. Physical examination of the nose revealed dark erythematous multiple papules that disappear on diascopy (Figure 2). This patient has a positive family history of increased centrofacial sweating (2 sisters and mother) but no visible lesions on the family members. No history of exacerbation with sunlight. The patient was treated with topical clindamycin and systemic doxycycline for 2 months but has not improved.

After discussing the potential side effects of the few treatment options available in our setup (cryotherapy and isotretinoin), and since even those options were only moderately effective and considering the lack of the relatively safe topical treatment options in our setting (topical indomethacin, tacrolimus, and botulinum toxin A), both patients were reassured considering the benign nature of the disease.

## 3. Discussion

GRN is a rare non-neoplastic autosomal dominant inherited disorder of the eccrine glands. There are also reports of autosomal recessive inheritance; however, the gene locus has not been identified [2].

Etiology and pathogenesis of this disorder have not been elucidated. A marked increase in sweat production on the nose occurs in granulosis rubra nasi, possibly resulting in a unique form of sweat retention. This hyperhidrosis precedes months or years before the development of maculae, papules, vesicles, pustules, and, later, telangiectasia. Excessive sweating may cause cystic dilatation of eccrine ducts and lead to the development of erythema and papules [4]. Eddowes had suggested that adenoids could be involved which can provide a source of irritation to the tip of the nose [5].

Heid et al. reported a case of pheochromocytoma in a 19-year-old woman with diffuse hyperhidrosis, tachycardia, and granulosis rubra nasi in whom surgical removal of the tumor was followed by the regression of nasal dermatosis [6].

Although the exact frequency is unknown, it is believed that men are affected more than women; likewise, 6 out of the 7 patients described by Jadassohna were boys [1]. Symptoms develop as early as 6 months but can occur at any age in childhood and occasionally in adults with the peak age being between 7 and 12 years.

It is clinically characterized by hyperhidrosis of the central part of the face, most conspicuous on the tip of the nose, followed by the appearance of dark erythematous papules that disappear on diascopic pressure, vesicles, and telangiectasia over the nose, cheeks, and upper lips. A few small pustules may occur. The tip of the nose is usually red/violet, cold, and not infiltrated. This condition is usually asymptomatic except for mild pruritus [7]. Heid et al. had reported an association with rhinorrhea [6].

At times, affected patients may have associated poor peripheral circulation and hyperhidrosis of the palms and soles [8]. Summer aggravation of lesions is occasionally found [8].

Granulosis rubra nasi usually resolves spontaneously at puberty; however, it occasionally persists indefinitely, in which case telangiectasia becomes the predominant feature [2].

The clinical picture of granulosis rubra nasi is distinctive, so there is rarely a problem with the diagnosis.

Histology is helpful in difficult-to-diagnose cases and demonstrates a perivascular and periductal mononuclear cell infiltrate, cystic dilatation of eccrine sweat glands, and vascular proliferation. Histopathology of a papule showed epidermal atrophy, basal vacuolar degeneration, and nodular collection of lymphocytes; few histiocytes; and multiple blood vessels with swollen endothelial cells [9].

Under polarized contact dermoscopy, Palit et al. reported a diffuse erythematous background with discrete round-to-oval pink and red structureless area, a few of them which showed overlying scattered to grouped brown dots [9].

Possible differentials include papulopustular rosacea, papular sarcoid, trichoepithelioma, and perioral dermatitis. Other differential diagnoses include acne vulgaris, lupus pernio, and lupus erythematosus.

It is advised to check for symptoms of pain and itching. Check for family history; the condition typically is autosomal dominant familial. Particular sensitivity to sunlight may suggest an alternative diagnosis. Other symptoms, such as a racing heart or a faint feeling, may indicate an alternative diagnosis (e.g., pheochromocytoma) [2], as diaphoresis associated with the excess catecholamine can potentially result in nasal hyperhidrosis and eventually present with GRN features.

To differentiate between these conditions, the distribution and morphology should be considered. The clinical features of perioral dermatitis consist of monomorphic small papules and pustules, erythema, and scaling, with distribution primarily around the mouth. In rosacea, there is erythema of the cheeks and nose, along with telangiectasia and accentuation of the erythema by vasomotor instability. Furthermore, in these two diseases, there is no hyperhidrosis of the central face, a prominent feature of granulosis rubra nasi.

Photosensitive dermatoses are usually easy to differentiate from granulosis rubra nasi because they are more extensive and do not show the characteristic hyperhidrosis [2].

Multiple eccrine hidrocystoma also presents with multiple translucent, dome-shaped papules on the periorbital and malar areas, with the frequent presence of craniofacial hyperhidrosis. However, this often occurs in middle-aged women and does not have the background erythema of the granuloma rubra nasi [10].

In the majority of cases, counseling and explaining the self-resolution nature of the condition after puberty are the mainstays of management. However, in rare cases, it occasionally persists indefinitely, and it can have significant cosmetic and psychological impact.

Treatments described in the literature, such topical indomethacin, tacrolimus, botulinum toxin A [4], cryotherapy, X-ray therapy, oral steroids, and tetracycline [11], have been tried with inconsistent outcomes.

Piotr and Katarzyna have described a 14-year-old patient that responded well to low-dose isotretinoin [12], and Kumar et al. reported that topical tacrolimus has been effective in decreasing vesicles in a 20-year-old patient who has had GRN [8].

## 4. Conclusion

GRN is a rare disorder. One should remember that it could be a complication of hyperhidrosis. Treatment is symptomatic and cosmetic. Counseling the patient about the self-limiting nature of the condition is of paramount importance. To my knowledge, this is the first time it has been reported in Ethiopian patients.

## Data Availability

No data were used to support the findings of the study.
